# Consistent Individual Behavioral Variation: The Difference between Temperament, Personality and Behavioral Syndromes

**DOI:** 10.3390/ani5030366

**Published:** 2015-07-08

**Authors:** Jill R. D. MacKay, Marie J. Haskell

**Affiliations:** Scotland’s Rural College, West Mains Road, Edinburgh EH9 3JGF, UK; E-Mail: marie.haskell@sruc.ac.uk

**Keywords:** personality, temperament, behavioral syndromes

## Abstract

**Simple Summary:**

The interchangeable usage of the words “personality”, “temperament” and “behavioral syndromes” in animal behavior research has often led to confusion. In this paper, we devise a framework for describing the behavioral phenomenon, betweenindividual/between-population variation, and between/across context variation. This framework can be used to give unique definitions of the three terms, supported by previous literature, giving clarity moving forward in the field of animal behavior.

**Abstract:**

Ethologists use a variety of terminology such as “personality”, “temperament” and “behavioral syndromes” almost interchangeably to discuss the phenomenon of individuals within a population of animals consistently varying from one another in their behavioral responses to stimuli. This interchangeable usage of terminology has contributed to confusion within the field of animal behavior and limits the study of the phenomenon. Here we use a rapid, non-exhaustive and repeatable search strategy literature review to investigate where there were unique distinctions between these three terms and where there was an overlap in their usage. We identified three main areas of confusion in terminology: historical usage which is not updated; a lack of precision between different fields of study; and a lack of precision between different levels of variation. We propose a framework with which to understand and define the terms based on the levels of variation ethologists are interested in. Consistent individual animal behavioral variation relates to the different structures of variation of between-individual/between-population and between and across contexts. By formalizing this framework we provide clarity between the three terms which can be easily defined and understood.

## 1. Introduction

The study of consistent individual behavioral variation in animals is complex with many concepts surrounding it. There is much diversity in how scientists from different fields discuss this variation and this is often thought to negatively impact the study of the phenomenon [1,2,3,4]. The aim of this review is to formalize how terms like personality, temperament and behavioral syndromes relate to the levels of behavioral variation we are interested in as ethologists. To do this we; (1) discuss what aspects of behavioral variation we are concerned with; (2) investigate how the terms have been used in the past using quantitative Web of Science searches; and (3) summarize the results of a literature review.

## 2. Basic Concepts in the Study of Individual Behavioral Variation

There are two important themes in the study of behavioral variation in animals. The first is that individuals may consistently vary from one another in the way they respond to stimuli or challenges. This variation is repeatable across time and possesses a heritable component [5,6,7], although this is not to imply that individual behavioural differences are entirely down to genetic factors. The variation is consistent within the animal, so it will show similar responses across time [8,9,10]. The second is that different traits may be linked across different contexts [11,12,13]. One pioneering study in this area found that sticklebacks which are more aggressive to intruders than others within the population during the breeding seasons, tend to be more aggressive to predators outwith breeding seasons [14]. This study established that individual sticklebacks varied consistently in the levels of aggression they showed under standard conditions, but also established that these differences were maintained across situations and time. This hints at the structure of behavioral variation mentioned above. It is easiest to understand this by constructing a simple thought experiment.

### 2.1. Behavioral Variation Has Structure

In this thought experiment, imagine that we discover a new species of animal. We gather individuals from this new species from the two different geographical locations that it occupies and bring them back to the research unit. We start to observe that there are non-random differences in their behaviors. Some researchers start to anecdotally describe certain individuals as “fearful” or “aggressive”. We begin to wonder if this can be quantified more scientifically in behavioral tests. It’s important to note that we are not directly measuring the animals’ internal feelings (there is no unit of fear) in these tests but rather interpreting their behaviors as indicators of their internal feelings [15].

In stage one of our investigations, we observe the behavior of all individuals in some test situations we have devised according to best practice in behavioural tests (reviewed well in [15]): response to a threat by an unfamiliar conspecific, response to a predator and response to a novel object. We find that each individual responds consistently to each of these tests and there is a range of responses across our population. At this stage we can conclude that the consistency shown means that these responses represent biological “traits” rather than the expression of more transitory “states” (in psychological terms).

In the second stage, we might examine how the animals respond in different versions of the same type of test: different novel objects, or a novel object and a novel human being, or a novel sound or different types of predator for instance. This would allow us to determine whether any response is general in scope or is only expressed in specific situations.

**Figure 1:**
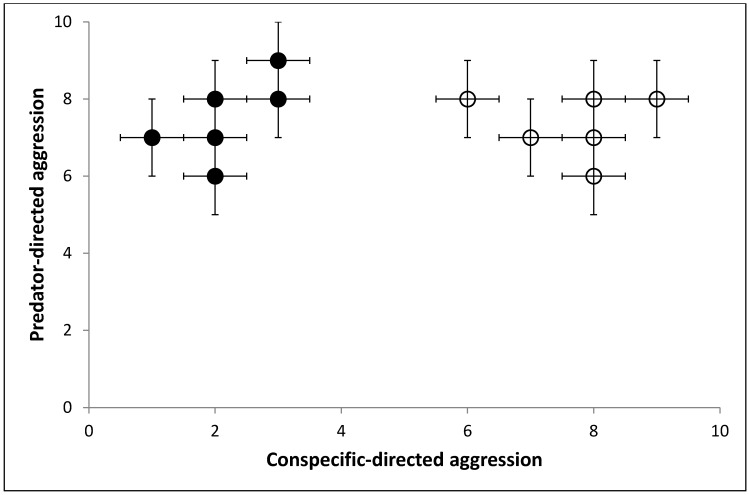
Chart showing the hypothetical scenario where two populations are given two behavioural tests, repeated multiple times. Population 1 is shown in black-filled circles and population 2 in hollow circles. Each point represents the mean score of an individual in their level of aggressive response to threat by unfamiliar conspecific *vs.* level of aggressive response to a predator (both on a scale of 1 (no response) to 10 (immediate and intense attack)), and the error bars the individual’s variability. Note relative consistency occurs within individuals, and there is a correlation between the two aggression tests in Population 1, but not Population 2.

Thirdly, we might compare the responses of the individuals across the different types of tests. We might discover significant correlations showing that animals that were bold in the novel object test were also aggressive towards intruders. However, on closer inspection of the data, we might find that there are different correlations between responses to the different tests in the two populations (populations in this sense referring to distinct groups of individuals from the same species [16] from different geographical areas). One population shows high levels of aggression to predators but low levels of aggression to conspecifics. The other population also shows high levels of aggression towards predators but high levels of aggression towards conspecifics (Figure 1). We discover that the first population comes from a habitat where food is evenly distributed and the population density is relatively low, with a sporadic threat of predators against the young. In the other population, food is more clustered, the population is denser and with the same predator threat. Having a strategy for actively defending food sources makes sense for the second population, whereas it is less important for the first population.

We propose that four distinct structures of variation (e.g., individual non-random variation from the mean) are depicted in this graph.

(1) Individuals vary from one another in the context of their response to a given test situation, *i.e.*, an individual datum on one axis which tells us how the individual animal will respond in this context only.
(2) Individuals vary from one another in multiple contexts, *i.e.*, the individual’s position on all possible axes, in this case it would be the x,y coordinates of any individual data point. This allows us to compare the whole of behavioural variation between individuals.
(3) There is population variation in one context, *i.e.*, the group’s spread of variation on one axis.
(4) There is population variation in multiple contexts, *i.e.*, the group’s spread of variation on all possible axes.

### Note on Dimensions of Variation

Figure 1 shows the results of two behavioural tests (predator related aggression and conspecific related aggression) but it could easily show any number of behavioral tests. In n-dimensional space we could completely map the individual’s response to every possible stimuli. We would see clusters of responses within individuals, as many behavioural tests are looking at the same underlying response, *i.e.*, response to novelty and response to startle are similar. By investigating where responses to behavioural tests cluster between individuals we could find underlying dimensions of variation within a population, *i.e.*, how the x axis is a structure of behavioral variation for the population in one context. In our example we only have two tests and so the underlying structures match the axis very closely. It is important to note that our axis is a way of quantifying the variation. In this instance, the x axis could be number of times an individual instigated a confrontation with a conspecific. The structure of variation which we might label ‘aggressiveness’ and is present within the whole population in that one context, is an unquantifiable scale which is correlated with, but not directly related to, the number of times the individual instigated confrontation. It is important to grasp that there is no unit of aggression, and the scale may not be a linear one (e.g., the difference in aggressiveness between 1 and 2 encounters may not be the same as the difference in aggressiveness between 13 and 14 encounters). With this caveat, the latent structure is still useful. We use it every day when saying an individual is more or less aggressive than the population norm. We use this scale to reference the individual to the population.

There is also the relationship of dimensions between populations, *i.e.*, the structure of variation for the population in multiple contexts. One population might show a relationship between two of the dimensions and another population might not. Therefore the structures of variation we are interested in relate both to context and individuals, between one context or multiple contexts, between individuals or between groups of individuals.

### 2.2. Commonly Used Terms

Now we have established there are different structures of variation, we can investigate how these structures are being studied across the many different disciplines of biology. The study of individual behavioral variation has been going on for many years and as a result there are many different approaches. In his cross-disciplinary review [17]  highlighted how the variety of terminology and usage in a variety of fields has negatively impacted the study of the phenomenon in animals:

“*A considerable number of publications on animal personality exist, but they are dispersed across a wide range of fields and are hard to find.*”

And this is echoed by [18]:

“*The impact that such [individual differences] have on behavior has only recently become of interest for behavioral and evolutionary ecologists. Two main reasons for this have been the lack of consistent terminology (e.g.*, “*personality*”, “*temperament*”, “*coping styles*” *and* “*behavioral syndrome*” *are all found in the literature) and the lack of ecological and evolutionary framework for temperament studies.*”

These reviews, and many more [1,2,3,4] share the general consensus that behavioral consistency exists in individuals, can affect the animal’s health, reproductive success, survival, welfare and productivity, and through the animal’s interactions affect the life of their conspecifics and nonconspecifics, e.g., human-animal interactions. Therefore it is an important aspect of animal behavior to study and one that, according to the aforementioned reviews, is being limited because of a lack of consensus in the usage of terminology. 

### 2.3. Associated Terms

Several words such as “context”, “trait” and “repeatability” are given specific meanings when discussed in relation to consistent individual behavioural variation. It is worth noting [11] distinction between a context (as a functional behaviour category) and a situation (a set of environmental conditions). Although [11] is a highly influential paper, the distinction in how they defined “context” and “situation” are not always upheld in the literature. If context is used in a manner to suggest a functional behaviour category it is usually stated as “feeding context”, “parental care context” *etc*. For the rest of this paper, we shall be utilising these associated terms as defined in Table 1 unless stated otherwise. 

As this paper has so far discussed consistent individual behavioural variation, and repeatability is a popular measure of consistency, it is worth briefly outlining what is meant by the term repeatability, a measure of consistency. Table 1 refers to repeatability as a measure of what proportion of the variation in the average phenotype can be explained by the differences between the individuals. This definition was taken from [19]. Most studies [8] utilise the variance method calculated as in [20] to estimate repeatability where:

$$Repeatability=\frac{variance\;among\;individuals}{variance\;within\;individuals+variance\;among\;individuals}$$

**Table 1:** Commonly used terms in behavioural variation.

Term	Meaning	Refs.
**Context**	A functional behavioural category such as “feeding”, “mating”, “parental care”, “contests”, *etc*.	[11]
**Situation**	A given set of environmental conditions at a certain point in time, e.g., high predator risk	[11]
**Trait (biology)**	Any empirical measure obtained from an individual, but not the theoretical concepts that are inferred from such measures.	[21]
**Repeatability**	Standardised measure of the differentiation in average phenotype across individuals, defined as the proportion of phenotypic variance explained by differences between individuals	[19]

Bell *et al*. [8] conducted a thorough meta-analysis of repeatability in behavioural studies in order to answer some fundamental questions about repeatability, such as: does it vary among age groups and does it decrease with the interval between observations? The main findings of [8] were that the average repeatability of a behavioural trait was 0.37 and that weighted for effect size across all estimates, repeatability was significantly greater than zero. As they put it, the data overwhelmingly supports the hypothesis that behaviour can be repeatable. There are many biologically valid reasons why an animal’s response to a situation might vary over time, and this is discussed in [9,10] but as behavioural plasticity is a topic worthy of several reviews within its own right, for the purposes of this framework we will assume that the hypothetical traits being referenced have been found to be repeatable with all appropriate measures taken to ensure this.

## 3. Quantitative Literature Search

Given the quantity of literature surrounding this topic and the high level of debate, we used a rapid review with a systematic and repeatable search process to ensure a transparent and repeatable selection process.

### 3.1. Refining Terminology

If all the terms mentioned above were truly synonymous, it would be easy to pick the terms used by the majority, or which was most useful, and to use them. Some researchers, however, maintain that there is a difference in the meanings of the terms. In the following section we will investigate why there is a tendency to use so many terms within the literature. We will demonstrate how recognizing and using the structures of variation, within individuals or populations and within one context or multiple contexts, helps to clarify the differences in the terminology.

Many different terms exist to discuss consistent individual behavioral variation, but not all these terms necessarily reference the structures of variation we are interested in: individual variation in one context, individual variation in multiple contexts, population variation in one context and population variation in multiple contexts. In this paper we focus on defining “behavioral syndromes”, “temperament” and “personality”. This is not to say that the other terms have no value, indeed “consistent individual behavioral variation” is a phrase that will be used repeatedly in this paper, but that for the purposes of formalizing a framework, these three terms are best suited.

First, it is necessary to understand what is meant by the three terms “behavioral syndromes”, “personality” and “temperament” as they are currently used in the literature. Web of Science is an important resource for researchers today, enabling the cataloguing and interrogation of vast numbers of scientific articles.

“Behavioral syndromes” are referred to most commonly as suites of correlated behaviors across situations or contexts which exist within populations [11,22,23], for example where activity and aggression correlate within some populations of sticklebacks but not others [12]. “Behavioral syndromes” are also referred to as existing within individuals [19], although [11] call this a behavioral type, and this apparent discrepancy of where the term sits in terms of levels of variation will be discussed later. “Personality” is most commonly defined as a style of behavioral response to a range of stimuli or situations and refers to individuals [24,25,26], whereas “personality dimensions” or “personality traits” are found in populations and species. An individual’s personality, or location on a personality dimension, must be consistent and repeatable [27,28]. “Temperament” is often referred to as the individual reaction to a challenging situation, or behaviours indicating affect [21,29,30], again with “temperament trait” being a little more general and referring to a population. With that being said, these summaries focus on highly cited papers, and there are disagreements within the literature, e.g., there have been discussions of “colony level personalities” in terms of ants [31] as opposed to calling these a behavioural syndrome as would have been defined in [22]. Further, temperament has been used in a broader sense, particularly in production animal sciences, and this too will be investigated further when discussing findings specific to temperament. This is yet another example of the disarray within and between different fields, and how adoption of a more standardized approach might improve the study of behavioral differences.

#### Search Process

To minimize variation in search results, all data was collected on 25 January 2013 through the creation of “citation reports” from Web of Science’s services. The following databases were searched: Sci-Expanded, SSCI, A&HCI and CPCI-S via Web of Science’s search field. The timespan ran between 1 January 1970 and 31 December 2012, although it should be noted that articles added to the repository post data collection date would not be included. In the “topic” field, each term was used to search for relevant articles. Lemmatization was on. For the term “behavioral syndromes”, the search string “‘behavioral syndrome’ OR ‘behavioural syndromes’ OR ‘behavioral syndrome’ OR ‘behavioral syndromes’” was used in addition to lemmatization to avoid searching for “behavior” and “syndrome” separately, after preliminary searches found this to be the most reliable method. Citation reports were created by year, by Web of Science category, by year by Web of Science category, by overlapping search terms.

### 3.2. General Findings

**Figure 2:**
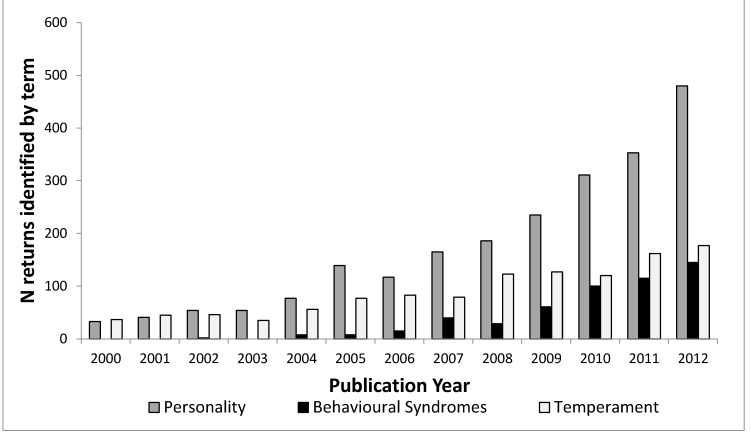
Number of articles per publication year for topic terms “personality”, “temperament” and “behavioral syndromes” in Web of Science categories agriculture, behavioral sciences, ecology, evolutionary biology, veterinary sciences and zoology.

**Figure 3:**
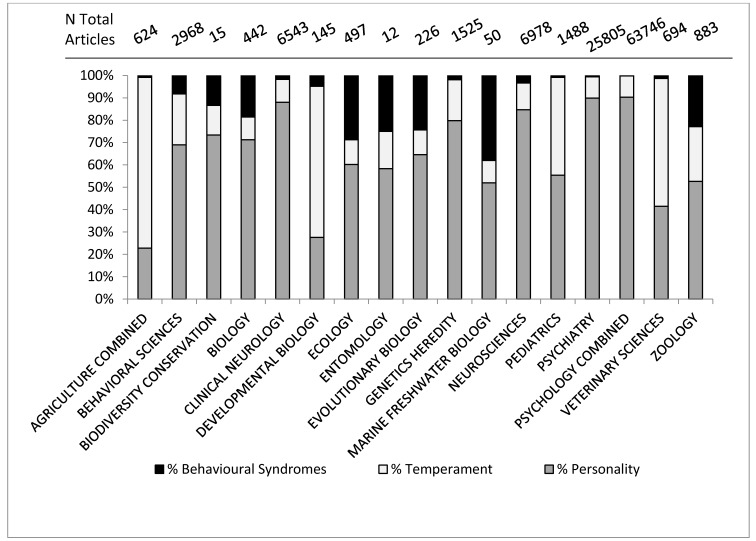
Percentage of articles in Web of Science category identified by the topic term “personality”, “temperament” or “behavioral syndrome”.

Figure 2 shows how the usage of all three terms has increased since the turn of the century. The search term “personality” identifies an ever increasing number of articles in strictly ethology-related fields over the past ten years; however, we can investigate this further. Web of Science uses its own categories (see Appendix 1) to group research into different fields. It is possible to investigate the distribution of articles that are identified by “behavioral syndrome”, “personality” and “temperament” across all these categories and this is done in Figure 3.

The first finding of note in Figure 3 is that the different fields do not use the terms to the same extent. Personality is the most predominant term overall and in the human-related fields, but does not dominate in the fields of agriculture, developmental biology, veterinary sciences, and to a lesser extent, pediatrics. In these fields temperament is the predominant term. Pediatrics and developmental biology are both fields concerned with early-life stages in humans, with developmental biology being more mechanistic, looking at specific mechanisms of cell, tissue and organism development, according to the definitions from Thomson Reuters (See Appendix 1). Behavioral Syndromes is most common in ecology, evolutionary biology and natural biology. The prevalence of the use of the term ‘temperament’ in pediatrics and human developmental sciences may be because of influential papers such as [32] which discuss temperament in the context of child development. The use of temperament is much lower within the fields of psychiatry and psychology compared to pediatrics, again reflecting its usage as describing child behavior, possibly before the “growth” of personality [29,30]. The confusion appears to stem from the rise in interest in consistent individual behavioral variation across fields, as demonstrated in Figure 2, but also because there have been some misconceptions and a lack of understanding of key points, not only between all three terms, but also within the usage of the terms themselves. We suggest three main reasons for confusion; the lack of precision between fields, the lack of precision between levels of variation and historical usage that has not adapted with advances in scientific understanding.

#### 3.2.1. Lack of Precision between Fields

Different fields do not utilize the terms in the same manner. Take the example of temperament. Within the literature, temperament is continually described as a basic trait, an underlying trait, an early-life trait or a trait with a biological origin [33,34,35,36] and very much in relation to an individual and the individual’s style of response to specific environmental challenges [37,38,39]. Hence it understandably has a large presence in the human-related early-life fields (Figure 3). However this explanation does not completely account for why temperament is so prevalent in agricultural or veterinary studies. In applied ethology fields, temperament is often used to refer to specific responses to tests, e.g., handling temperament [4], or how an individual reacts to novel or challenging situations [40]. In applied animal sciences, temperament is often measured in units or with an ethogram (see Section 3.5) which makes it easier to investigate research questions such as heritability of temperament [41,42], despite the personality constructs themselves being more complicated than simple genetic determinants can account for [43]. In some ways, applied animal science has used temperament as a simpler, more easily quantifiable approach to behavioral variation, but not necessarily always recognizing the unquantifiable nature [43] of a personality construct. We do not expect that farm animals are more childlike than animals studied in other contexts. The two study areas utilize the same term in slightly different ways, with animal related fields often using a different term to one that would be suitable in the fields of psychology, often because of fears of anthropomorphism [25] which we will come to later.

Behavioral syndromes are often stated as being analogous to personality [22] or animal personality [44,45,46], or a personality dimension [47]. This is an interesting idea, suggesting that behavioral syndromes and personality are comparable (but importantly, not the same) in certain respects. It is not surprising, nor wrong, that when new terms are introduced into the literature they are accompanied by an existing term, more familiar to the audience, to aid in understanding. It is important to note however that “personality” and “animal personality” have been considered two separate things by psychologists and are not analogous themselves, with conceptual misunderstandings giving rise to the idea that the common usage of “animal personality” is the same as how psychologists use “personality” [21]. Uher argued that ethologists use “animal personality” more similarly to how a psychologist uses the term “personality dimension”, *i.e.*, a structure of behavioral variation within a population. Furthermore, a psychologist would certainly not consider a “personality” equivalent to a “personality dimension” [48]. Hofstee (1994) suggested that a personality dimension should be considered a “point of reference” for the study of the individual’s personality as a whole, that dimensions should be considered as a way of scaling variation within a population. An individual can only be at a high extreme of the scale in comparison to the distribution of individuals along that scale. In practical terms the dimension of “boldness” allows me to declare an animal “bold” in comparison to the rest of the population, implying there are less “bold” individuals in the population. From this we can see there is ready adoption of terms across fields but not always a clear understanding of particular nuances in certain fields, creating further confusion between the terms in the field of applied animal behavior.

#### 3.2.2. Lack of Precision between Levels of Variation

Another confusing issue is the difference in the use of terms to describe behavioral variation shown between individuals and the behavioral variation shown between populations. In the previous section, the difference between “personality” and “personality dimension” was mentioned. They could be regarded as two different levels of variation, the personality referring to the individual and the personality dimension referring to the population.

Personality dimensions occur in a set of individuals and are a construct used to describe the range of behavioral variation in a cluster of similar responses at the context level relating to the same internal state [21,49,50], e.g., the human Five Factor Model (FFM) of personality attempts to describe the human population across cultural and sociological boundaries using the five dimensions of neuroticism, agreeableness, extraversion, openness and conscientiousness (although the number of dimensions warrants further discussion below). Thinking back to the thought experiment in the previous section, a personality dimension would be similar to an axis of variation, but the individual animals, or data points, give no information about the range of the axis. Only by measuring a population can the dimension be characterized. In contrast, personality is the sum of those dimensions in the individual [21,29,48,51], or the unique coordinates of any individual in multi-dimensional space. So how can a behavioral syndrome be similar to both a personality and a personality dimension when these two things are referencing different levels of variation? To further confuse the matter the common definition of behavioral syndromes implies that a behavioral syndrome refers to more than one behavioral trait, whereas a personality dimension is singular. Therefore the common analogy made between personality or personality dimensions and behavioral syndromes is very misleading and fundamentally confuses variation at the individual level with variation at the population level.

#### 3.2.3. Historical Usage

There is a final source of confusion and that is the case of historical usage. In applied ethology, “temperament” is used because farmers used it to refer to their animals [52] and there has been speculation that the use of the term “personality” has been avoided because of fears of anthropomorphism [25]. Behavioral syndromes may also suffer from a similar problem in our anecdotal experience. When writing this paper, we received many personal communications from ethologists who felt that the term “behavioral syndromes” implied that the structure of behavioral variation in animals was part of a disease or ailment, most likely due to the connotations of the word “syndrome”. We did not find any animal-based papers which made this connection. However, the field of neuroscience does use the term “behavioral syndromes” to mean a suite of behaviors associated with disease symptoms, such as in Alzheimer’s or psychopathies [53,54,55] and these articles can be found while using behavioral syndromes as a keyword if not searching in the specific fields related to animal behavior. This historical “baggage” linked to each term will be discussed in more detail in the specific findings of the literature review in each term, but it draws attention to the desperate need for a framework with which to discuss this behavioral variation.

### 3.3. Specific Findings: Behavioral Syndromes

**Figure 4:**
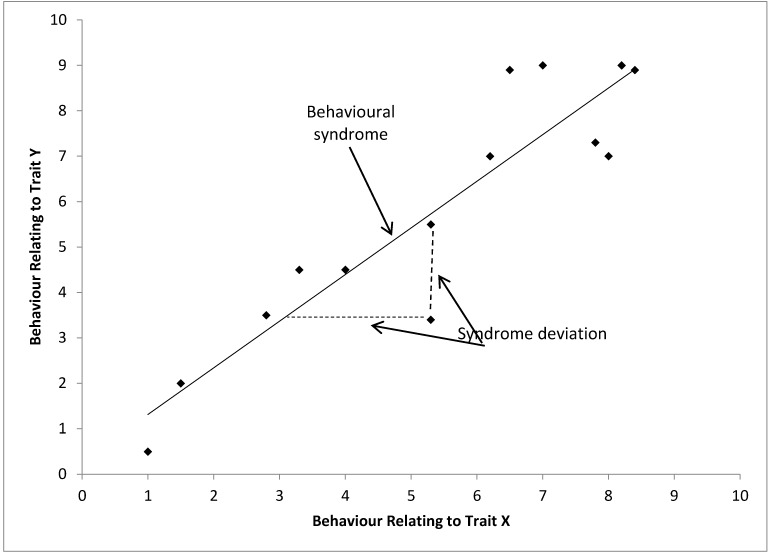
Schematic representation of a behavioral syndrome (correlation) between behavioral traits expressed by a set of individuals. Each point represents a hypothetical individual, adapted from [13,22].

Bell (2007) represents a behavioral syndrome diagrammatically and this definition (adapted in Figure 4) has been used by many researchers including in a set of recent papers debating how behavioral syndromes should be considered statistically and in meta-analyses [13,44,56]. Here it is clear that in this definition the behavioral syndrome is a function of the relationship of two different dimensions within the population and can only be seen in the set of individuals. Therefore it would be inappropriate to consider a behavioural syndrome in an individual without using a different definition of “behavioural syndrome”, which would be comparable to definitions of personality (see below). This dilutes the importance of “behavioural syndromes” in a population. Behavioral syndromes refer to multiple contexts, *i.e.*, two axes of variation in this case, and multiple individuals, *i.e.*, in this case it is a function of the relationship between the two dimensions in the population. It would therefore be a high level term, referring to variation between contexts and populations and not referring to within individual behavioral variation. It is not always clear when authors are using behavioral syndromes on the individual rather than the population level, and this is a particular cause of confusion within the usage of behavioral syndromes. This diagrammatic depiction of behavioral syndromes is similar to our earlier thought experiment, and forms the basis of our final framework later.

### 3.4. Specific Findings: Personality

As previously mentioned, an individual’s personality is the sum of their locations on the personality dimensions present within the population [21,49,50]. This returns to the important difference between a personality (which is at the level of the individual across many contexts) and the personality dimension (which is at the level of the population in one context). This difference is mostly upheld by the literature, particularly in those species where personality is used frequently, *i.e.*, dogs [57,58,59].

The Five Factor Model is a popular and widely known personality model [17,25] and predicts that an individual will act in a certain manner, e.g., boldly, but not in quantifiable units. However [25] note that in animals, many of the dimensions are contracted or absent. Does restricting the number of personality dimensions available to the personality model change the definition of personality? Some researchers have worked on meta-analyses of behavioral studies and suggested that animal behaviors can be explained by two factors, activity-exploration and fear-avoidance [60]. This is conceptually very similar to two dimension models of temperament proposed by [61] where behavioral variation is split into two dimensions, the quantitative activity (high/low) and qualitative (active coping/passive coping) dimensions. Contracting the personality model does not change how we would describe it. It is still a model of behavioral variation referring to the individual in more than one context. One could argue that the active/passive models are incorporated into the term “personality” as two-dimensional models as opposed to the more complex models in human psychology such as the FFM, Belbin’s Team-Role Model.

#### Personality *vs*. Temperament, the Anthropomorphism Problem

When giving an introduction to the idea of “personality” in animals [17] grouped together “personality” and “temperament” results, in part because the research had already done so, and partly because he noted that “temperament” appeared to be used in substitution for “personality” due to fears of anthropomorphism. There is no doubt that this has contributed a great deal to the conflagration between temperament and personality in ethology. This idea of “personality” being “of a person”, *i.e.*, solely in the human domain, was widely accepted in science several decades ago with one human psychology review saying:

“*When we apply the notion of personality in order to characterize animal behavior we tend to use it parenthetically. This is not so with temperament, a concept applied to characterize both human and animal populations.*”.

[62](p. 113)

In the same review, Strelau also considered that combining the terms “temperament” and “personality” (in humans) made it impossible to differentiate the variance associated with only one. He argued that personality encompasses social environment, the feedback that behaviors will generate from other people, e.g., that an aggressive person will be received differently to a non-aggressive person. Strelau argued that many definitions of temperament do not make this distinction. Similarly when discussing child development, [29] (p. 207) included the idea that:

“*temperament and experience “grow” a personality*”.

If we can use the word personality in animal behavior research, should we not also recognize and make use of the different influences on behavior referred to by these terms? In the general findings we saw in Figure 2 and Figure 3 that personality has increased in popularity throughout the years and is increasingly used throughout all scientific fields interested in animal behavior.

Anthropomorphism is often defined as the attribution of human characteristics and states (thoughts, feelings, motivations and beliefs) to nonhuman animals [63,64,65]. From this statement arises a discussion that can suffocate the study of individual behavioral variation in animals, creating fear and spawning new word usage, the so-called “specter of anthropomorphism” [25]. It is not anthropomorphic to say there are non-random patterns of behavior in individual animals. The interpretation of those states (e.g*.*, to say the non-random pattern is one of fearfulness) is anthropomorphic in that it attributes a characteristic of humans to animals, although not inappropriately. If the definition of personality relies on a theory of mind it would be anthropomorphic to ascribe personality to animals. If the definition of personality relies on the non-random patterns of behavior that allow prediction of behavior in different contexts, anthropomorphism is not so much of a concern. The argument against using the term “personality” in animal research also ignores the fact it is already a term commonly used in reference to animal behavior. Therefore, fears of anthropomorphism should not be a reason for researchers to avoid using the term “personality” in future, particularly if “personality” is defined in reference to structures of behavioral variation, as we propose.

### 3.5. Specific Findings: Temperament

Let us return to the question of why temperament is so prevalent in fields not related to early life and development (Figure 3). Firstly, “temperament testing” has recently undergone some intensive research in agriculture and borrows many of the words from the personality literature [66] such as bold/shy. A temperament trait is similar to the personality dimension of the animal’s response to a specific context, but we tend to be interested in broader personality dimensions, e.g., fearfulness as opposed to fearfulness in situation x. Therefore temperament refers to the one-context level of behavioral variation. It differs from personality dimensions in its specificity of context and should correlate with the broader personality dimension the context relates to. Many behavioral assessments of welfare adapt some form of temperament test [67,68] because the consistency and repeatability of a temperament trait allow for comparison over time. While this borrowing of terminology has undoubtedly helped to confuse the issue, it has given temperament a second aspect. Temperament in these cases is measured in a specific context, similar to the early, more qualitative work done discussing the behavior of stock with the stockpeople who manage them [69]. Early usages of temperament in agricultural sciences relate the animal’s behavior to specific contexts, for example “milking temperament” or “handling temperament” in cattle [52]. This context is sometimes quantified, *i.e.*, the speed of escape from a handling crate. In this case an additional reference for the behavioral variation is given, speed. Temperament can therefore reference behavioral variation in the individual as well as the population, as by giving a unit of variation, it does not need to reference the underlying personality dimension. For example, if a beef steer leaves the handling pen at 3 m·s^−^^1^ we can relate this more biologically based trait to the underlying dimension of fearfulness. This is different from personality, which refers more to the underlying structure of variation we cannot quantify. By incorporating facets of genetic inheritance and physiological mechanisms of behavior, predicting testable results in realistic situations, temperament occupies a niche which personality and behavioral syndromes cannot fill. 

## 4. A Framework for Future Reference

We have seen how these three terms, “behavioral syndromes”, “personality” and “temperament” could relate to the different structures of between-individual/between-population and between and across contexts, but can we formalize this in a framework?

**Figure 5:**
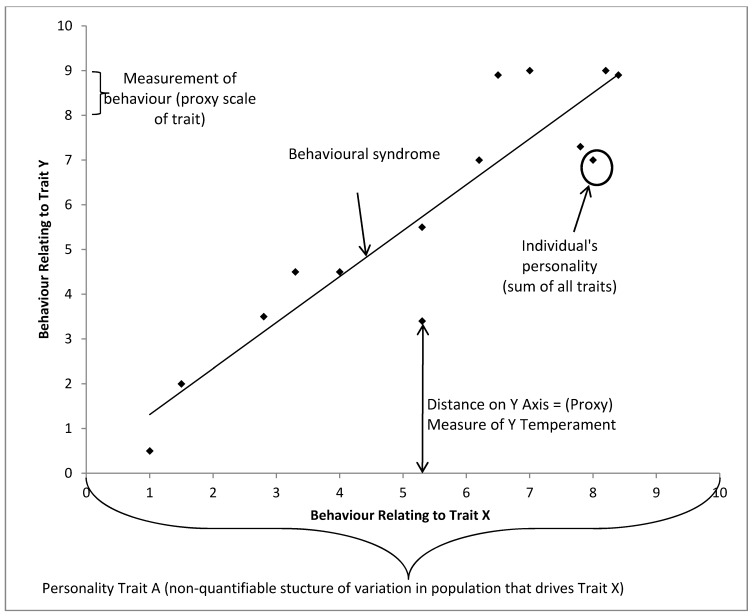
Proposed framework of behavioral syndrome, personality and temperament in a hypothetical population with only two structures of behavioral variation, each point represents an individual.

Confusion between personality and temperament almost certainly stems from the long history of wariness regarding anthropomorphism. We feel this has helped to further blur the overlap between the terms temperament and personality in ethology. Methodological problems concerning the measurement of single traits has helped to confuse the issue further. The concept of behavioral syndromes, while addressing a new approach to consistent individual behavioral variation, has suffered from attempting to include both personality and temperament without an appropriate framework or referencing the psychological meanings, but clearly references the structure of the relationship between personality dimensions rather than the dimensions themselves. So how do we use these three terms constructively? In order to choose the appropriate terminology, we feel the researcher must ask themselves what their model of behavioral variation is predicting (*i.e.*, population behavior, individual behavior or patterns in behavior between multiple contexts). In our opinion, by referring to the benefits and limitations of each model, researchers can select the most appropriate model for their study. We propose a framework as illustrated in Figure 5, which is to our knowledge the first time the three terms have been defined in reference to one another, and with unique aspects. It is our belief that the adoption of this framework would be useful and bring clarity to the field, however its robustness in practice will only be made clear with time and we would welcome future revisions to the framework if future work changes the field of study.

In Figure 5, we can see that individual behavioral variation needs to be referenced to the population as a whole, with each personality dimension driving variation in certain observed behaviors. As we only have the capacity to record behavior as an indicator of the animal’s emotional state, the individual’s behaviours can act as a proxy measure of the underlying trait (e.g., temperament), which closely mirrors the underlying personality trait that may be uncovered through exploring underlying structures of variation in the data. The individual’s location on *n* dimensions is its personality, and a reductive personality model, such as the Five Factor Model, would describe the individual’s location on five dimensions. Finally, by comparing a population’s spread on two or more traits we can describe behavioural syndromes which occur.

### 4.1. A Working Definition of Personality

In an ideal world, to understand an individual’s behavioral reactions, it would be tested with every possible stimuli and all its reactions would be recorded. Additionally, in this hypothetical situation, the effects of all habituation and learning events are known. The individual would then have a location in n-dimensional space, for n reactions to n stimuli, and this would be a perfect model for predicting that individual’s behavior, assuming the individual received no extra “experience” with which to modify its behavioral responses. Similar stimulus would elicit similar reactions. For example, a loud noise created by a siren shares many properties with a loud noise created by an explosion and we would expect a similar reaction to both from the same individual. If we then measured all other individuals in the population, we would have a model describing all the behavioral variation present and noise in that population. Where a distribution of responses exists to the same kind of stimulus that cannot be explained by other factors such as sex, age, *etc*., a personality dimension exists within the population, and an individual animal has a position along that dimension within that distribution.

This, of course, cannot be done. Therefore, we roughly characterize the behavior of the individual to a small set of stimuli, placing the individual’s behavioral response in the context of the responses of the rest of the population. Personality describes the individual’s behavioral variation in reference to the personality dimensions found in the population.

### 4.2. A Working Definition of Temperament

Temperament is perhaps the most difficult term to define thanks to its long history of usage in the separate fields of pediatrics and agricultural sciences. Temperament is the animal’s behavioral response in a single context measured on some biological scale, such as flight speed. This definition is of more use to ethologists, who often find themselves using proxy measures and can make comparisons across populations when faced with the same context. It is this ability to quantify which makes temperament so useful, as this can be tested several times, does not require much *post-hoc* conversion to be understood as a dimension (*i.e.*, through factor analysis, as in personality dimensions) and can be related to other biological measures more easily. However, the compression of temperament test results into bivariate categories (such as good/bad) can lead to a loss of information.

In essence, this view of temperament places the individual’s behavioral response in context of a certain situation and is associated with variation in the underlying personality dimension within the population. For example, we talk about handling temperament, in which between-individual variation is likely motivated by the animal’s underlying fearfulness when handled. This definition of temperament allows us to say that when separated from a group, the individual has a return latency of n seconds. We can use this to estimate how the animal will react to other social stimuli, but it is a measure which likely covaries along a personality dimension, not a measure of the dimension itself. Temperament is the ability to describe the animal’s behavior in units and is used as a proxy for the non-quantifiable aspects of personality.

### 4.3. A Working Definition of Behavioral Syndromes

Behavioral syndromes are most useful when used to describe variation within populations, not individuals. Behavioral syndromes link two or more dimensions across a population and may imply that these clusters do not allow for complete plasticity within a population. They are a description of the distribution of personalities within a population. When defining personality, we discussed where clusters may form in a population, represented by the concept of personality dimensions present within the population. The behavioral syndromes term places the differences between individual personalities in an evolutionary context within multiple dimensions and populations (although that is not to say the behavioural syndromes are the only term referring to an evolutionary context). Behavioral syndromes reference both contexts and other populations and their behavioral variation. Another way of describing the individual personality could be its deviation from what the behavioral syndrome predicts. They make good predictions of the behavior of a group of individuals, and can be thought of as “high level” modelling.

## 5. Conclusions

Through a rapid systemic literature review, we have investigated the uses and changes of terminology surrounding individual behavioural variation in the literature. We have outlined our argument for the unique definitions of temperament, personality and behavioral syndromes based on a framework that recognizes different levels of behavioral variation. We consider that the use of such a framework would aid the study of behavioral variation through the recognition of the levels of variation, and whether behaviours are being used as proxy traits, or constructs being described through *post-hoc* testing, and how these considerations affect the study of animal behavior.

## Appendix 1

Science Citation Index Expanded category selected definitions from Thomson Reuters. (http://ip-science.thomsonreuters.com/mjl/scope/scope_scie/).

**Table:**

**Science Citation Index Expanded Category (N Total Article Returns)**	**Defintion**
**AGRICULTURE, DAIRY AND ANIMAL SCIENCE Combined with AGRICULTURE, MULTIDISCIPLINARY (*n* = 427)**	Agriculture, Dairy and Animal Science covers resources on the selection, breeding and management of livestock, including animal science, animal nutrition, poultry science, animal breeding and genetics, dairy science, and animal production science. Agriculture, Multidisciplinary covers resources having a general or interdisciplinary approach to the agricultural sciences. Regional and multi-subject resources are also covered.
**BEHAVIORAL SCIENCES (*n* = 1941)**	Behavioral Sciences covers resources dealing with the biological correlates of observable action in humans or animals. These include aggression, sexual behavior, and learning as well as the various factors, natural or pharmacological, that alter such behaviors. Resources in this category cover neurobiology, experimental psychology, ethology, cognitive assessment, and behavioral consequences of neurological disorders.
**BIODIVERSITY CONSERVATION (*n* = 9)**	Biodiversity Conservation covers resources on the conservation management of species and ecosystems. Topics include conservation ecology, biological conservation, paleobiology, natural history and the natural sciences.
**BIOLOGY (*n* = 268)**	The Biology category includes resources having a broad or interdisciplinary approach to biology. In addition, it includes materials that cover a specific area of biology not covered in other categories such as theoretical biology, mathematical biology, thermal biology, cryobiology, and biological rhythm research.
**CLINICAL NEUROLOGY (*n* = 4623) ***	Clinical Neurology covers resources on all areas of clinical research and medical practice in neurology. The focus is on traditional neurological illnesses and diseases such as dementia, stroke, epilepsy, headache, multiple sclerosis, and movement disorders that have clinical and socio-economic importance. This category also includes resources on medical specialties such as pediatric neurology, neurosurgery, neuroradiology, pain management, and neuropsychiatry that affect neurological diagnosis and treatment.
**DEVELOPMENTAL BIOLOGY (*n* = 110)**	Developmental Biology includes resources focused on the specific mechanisms of cell, tissue, and organism development, as well as gametogenesis, fertilization, biochemistry and molecular genetic control of development, cell biology of gametes and zygotes, and embryology.
**ECOLOGY (*n* = 285)**	Ecology covers resources concerning many areas relating to the study of the interrelationship of organisms and their environments, including ecological economics, ecological engineering, ecotoxicology, ecological modeling, evolutionary ecology, biogeography, chemical ecology, marine ecology, wildlife research, microbial ecology, molecular ecology, and population ecology. This category also includes general ecology resources and ones devoted to particular ecological systems.
**ENTOMOLOGY (*n* = 10)**	Entomology covers resources concerning many aspects of the study of insects, including general entomology, applied entomology, regional entomology, apidology, aquatic insects, insect biochemistry and physiology, economic entomology, integrated pest management, environmental entomology, and pesticide science.
**EVOLUTIONARY BIOLOGY (*n* = 130)**	Evolutionary Biology covers resources concerning the molecular, natural selection, and population mechanisms of evolution; the evolution of species and related groups; the classification of organisms based on evolutionary relationships; and the biology and ecology of extinct organisms.
**GENETICS AND HEREDITY (*n* = 898)**	Genetics and Heredity includes resources that deal with the structure, functions, and properties of genes, and the characteristics of inheritance. This category also considers heritable traits, population genetics, frequency and distribution of polymorphism, as well as inherited diseases and disorders of the replicative process. The category is distinguishable from Biochemistry and Molecular Biology by its specific emphasis on the gene as a single functional unit, and on the gene’s effect on the organism as a whole.
**MARINE FRESHWATER BIOLOGY (*n* = 30)**	Marine and Freshwater Biology covers resources concerning many aquatic sciences, including marine ecology and environmental research, aquatic biology, marine pollution and toxicology, aquatic botany and plant management, estuarine and coastal research, diseases of aquatic organisms, molluscan and shellfish research, fish biology and biofouling.
**NEUROSCIENCES (*n* = 4469) ***	Neurosciences covers resources on all areas of basic research on the brain, neural physiology, and function in health and disease. The areas of focus include neurotransmitters, neuropeptides, neurochemistry, neural development, and neural behavior. Coverage also includes resources in neuro-endocrine and neuro-immune systems, somatosensory system, motor system and sensory motor integration, autonomic system as well as diseases of the nervous system.
**PEDIATRICS (*n* = 1165) ***	Pediatrics covers resources on basic and clinical research in pediatrics. Numerous pediatric specialties are covered including, cardiology and respiratory systems, dentistry, dermatology, developmental behavior, gastroenterology, hematology, immunology and infectious diseases, neurology, nutrition, oncology, psychiatry, surgery, tropical medicine, urology, and nephrology. Coverage also includes perinatology, neonatology, and adolescent medicine.
**PSYCHIATRY (*n* = 18785) ***	Psychiatry covers resources on clinical, therapeutic, research, and community aspects of human mental, emotional, and behavioral disorders.
**PSYCHOLOGY Subcategories: Applied; Biological; Clinical; Developmental; Educational; Experimental; Mathematical; Multidisciplinary; Psychoanalysis; Social (*n* = 50259) ***	Psychology is concerned with resources on the study of human behavior and mental processes. This category covers the biological and neurological underpinnings of perception, thought, and behavior; psychological development and change over the life span; in addition to emotional and mental disturbances and diseases and their treatment. Resources that report on animal behavior to illuminate human behavior and mental processes are also covered.
**VETERINARY SCIENCES (*n* = 526)**	Veterinary Sciences covers resources concerning both the research and clinical aspects of animal health, diseases, injuries, nutrition, reproduction, and public health. This category includes materials on companion, farm, zoo, laboratory, wild, and aquatic animals.
**ZOOLOGY (*n* = 504)**	Zoology covers resources concerning a broad range of topics on the study of animals. This category ranges from animal behavior and animal physiology to some aspects of animal ecology. The category does not include veterinary medicine, ornithology, or most aspects of entomology.

***** These subjects are predominantly human based, however may feature animal species as a model or be used in comparative biology.
